# Exploring the potential of pheophorbide A, a chlorophyll-derived compound in modulating GLUT for maintaining glucose homeostasis

**DOI:** 10.3389/fendo.2024.1330058

**Published:** 2024-03-11

**Authors:** Saptadipa Paul, Anuma Pallavi, Nikhil R. Gandasi

**Affiliations:** ^1^ Cell Metabolism Lab (GA-08), Department of Developmental Biology and Genetics (DBG), Indian Institute of Science (IISc), Bengaluru, India; ^2^ Department of Medical Cell Biology, Uppsala University, Uppsala, Sweden

**Keywords:** pheophorbide A, GLUT1 trafficking, molecular docking, cell viability, live cell imaging, molecular dynamics simulation

## Abstract

**Introduction:**

Pheophorbide A, a chlorophyll-breakdown product, is primarily investigated for its anti-oxidant and anti-inflammatory activity. Recent reports on pheophorbide A have shown its potential in lowering blood glucose levels, thus leading to the exploration of its use in diabetes management. Literature has also shown its effect on enhanced insulin secretion, whereas its mechanism on glucose stimulated insulin secretion (GSIS) in pancreatic β cells remains unexplored.

**Methods:**

*In-silico* and *in-vitro* investigations were used to explore the effect of pheophorbide A on class I glucose transporters (GLUTs). *In-silico* studies include - Molecular docking studies and stability assessment using GROMACS. *In-vitro* studies include - MTT assay, Glucose uptake assay, Live-cell imaging and tracking of GLUTs in presence of Pheophorbide A compared to control.

**Results:**

Molecular docking studies revealed better binding affinity of pheophorbide A with GLUT4 (−11.2 Kcal/mol) and GLUT1 (−10.7 Kcal/mol) when compared with metformin (−5.0 Kcal/mol and −4.9 Kcal/mol, respectively). Glucose levels are largely regulated by GLUTs where GLUT1 is one of the transporters that is ubiquitously present in human β cells. Thus, we confirmed the stability of the complex, that is, pheophorbide A-GLUT1 using GROMACS for 100 ns. We further assessed its effect on a pancreatic β cell line (INS-1) for its viability using an MTT assay. Pheophorbide A (0.1–1 µM) showed a dose-dependent response on cell viability and was comparable to standard metformin. To assess how pheophorbide A mechanistically acts on GLUT1 in pancreatic β cell, we transfected INS-1 cells with GLUT1–enhanced green fluorescent protein and checked how the treatment of pheophorbide A (0.50 µM) modulates GLUT1 trafficking using live-cell imaging. We observed a significant increase in GLUT1 density when treated with pheophorbide A (0.442 ± 0.01 µm−2) at 20 mM glucose concentration when compared to GLUT1 control (0.234 ± 0.01 µm−2) and metformin (0.296 ± 0.02 µm−2). The average speed and distance travelled by GLUT1 puncta were observed to decrease when treated with pheophorbide A. The present study also demonstrated the potential of pheophorbide A to enhance glucose uptake in β cells.

**Conclusion:**

The current study’s findings were validated by in-silico and cellular analyses, suggesting that pheophorbide A may regulate GLUT1 and might be regarded as a potential lead for boosting the GSIS pathway, thus maintaining glucose homeostasis.

## 1 Introduction

Diabetes mellitus (DM) is an endocrine metabolic disorder characterized by high blood glucose levels, resulting in impaired insulin secretion/action. Maintenance of glucose homeostasis is critical for treatment of hyperglycemia (1). Glucose-stimulated insulin secretion (GSIS) in the single-pancreatic β cells (2) is initiated by entry of glucose via glucose transporters (GLUTs) (3). GLUTs help the facilitative transportation of glucose across the cell membrane, and there are 14 different types classified into four groups. Among them, GLUT1’s high-expression levels (Km value of ~1–5 mM) make it one of the predominant transporters in human pancreatic β cell and, hence, are vital for glucose uptake (4–6). As a result, understanding the role of GLUTs, particularly GLUT1, in β cells and how they contribute to glucose metabolism may lead to more effective therapeutic approaches for treating DM.

Recent reports have shown G protein–coupled receptors (GPCRs) as important drug targets for treatment of DM. For example, GPR40-bound free fatty acid ligands may upregulate lipotoxicity in the MIN6 cells (7, 8). Recently, glucagon-like peptide 1 (GLP-1) and GLP-1R agonists are widely used for DM treatment although GLP-1 has a half-life of 2 min and undergoes rapid degradation by dipeptidyl peptidase 4 and endopeptidase, although stable forms may prevent this quick degradation. Few studies have also suggested GLP-1’s possible association with certain tumors such as malignant thyroid neoplasms, pancreatic neoplasms, and so forth (9, 10). To counteract these limitations, GLUTs might serve as a better target in DM since it acts via GSIS. A broad range of current therapies includes oral hypoglycemic agents, sometimes in combinations, but prolonged use of these are related to adverse side effects (1). Therefore, medicinal herbs and their combinations are considered as an alternative source for treating DM, which might be devoid of such side effects. Madhurakshak active (MA), a commercial anti-diabetic polyherbal formulation, composed of *Momordica charantia*, *Syzygium cuminii*, *Mangifera indica*, and *Gymnema sylvestre*, is widely used in the management of DM. Previous studies have showed its potential in management of DM (11). Additionally, liquid chromatography–mass spectrometry (LC-MS/MS) analysis of MA in its major peak showed presence of pheophorbide A, which is a chlorophyll-breakdown product (12). Pheophorbide A is primarily investigated for its anti-oxidant and anti-inflammatory activity; it has the potential to lower blood glucose levels, thus could be explored for its use in diabetes management. Studies show that it could enhance insulin secretion and manage high-glucose levels during hyperglycemia (13–15), although its mechanism of action in pancreatic β cells is not known. Previous studies on various plant extracts and a few bioactive compounds have reported their anti-diabetic properties with respect to increasing GLUT expression (16–18). Considering that the expressed GLUTs must be trafficked and functional for effective glucose absorption, expression alone is insufficient. It remains unexplored how these bioactive compounds, such as pheophorbide A, drive GSIS via GLUTs in pancreatic β cells. Cell-based bioassays are critical for this purpose and have been employed with some of the natural product formulations (19). Considering the same, this study is aimed at evaluating the possible mechanisms of pheophorbide A in GSIS via GLUT1 using *in-vitro* and *in-silico* approaches.

## 2 Materials and methods

### 2.1 Materials

Pheophorbide A (Purity:≥90%) was procured from Santacruz Biotechnology Inc. Stock solution of pheophorbide A at 1(phosphate-buffered saline):0.000037(dimethylsulfoxide, DMSO) was prepared on the day of experiment. All other chemicals used in the study were of analytical grade. The software and server used for *in-silico* analysis are PubChem, protein data bank (PDB) database, SWISS ADME server, ProTox-II, PyRx software that was used by AutoDock Vina, and GROMACS 4.0.6.

### 2.2 *In-silico* drug-likeness analysis

The drug-like and toxicology properties of the selected compound were assessed by using the following web server: SwissADME, Pub-Chem, and ProTox-II.

#### 2.2.1 Physicochemical, Lipinski rule, and ADME properties

To assess the selected compound physicochemical, Lipinski rule, and ADME properties, the associated SMILES of the compound were taken from PubChem database (www.pubchem.ncbi.nlm.nih.gov). Followed by loading the same in SWISS ADME server (http://www.swissadme.ch/index.php) (20, 21).

#### 2.2.2 ProTox-II analysis

The toxicity profile of the compound was assessed based on 33 models, that is, identification of several toxicity endpoints such as acute toxicity, hepatotoxicity, cytotoxicity, carcinogenicity, mutagenicity, immunotoxicity, adverse outcomes (Tox21) pathways, and toxicity targets of the selected compounds were computed using ProTox-II (tox.charite.de) (21). The associated SMILES of the compound were taken from PubChem database (www.pubchem.ncbi.nlm.nih.gov) and loaded in Tox prediction link on ProTox-II server, followed by selecting the toxicity profile of interest and initiating Tox prediction.

#### 2.2.3 Protein preparation

The three-dimensional (3D) structure of the proteins such as GLUT1 (4PYP) and GLUT3 (7SPT) was derived from PDB whereas, for GLUT2 and GLUT4 was taken from AlphaFold. The downloaded structures are complexed with peptide inhibitors, water molecules, other inhibitors, and heteroatoms. The proteins were prepared for docking using Discovery studio visualizer by removing the peptide inhibitors, water molecules, other inhibitors, and heteroatoms from the protein complex. This modified protein molecule was used for docking (21, 22).

#### 2.2.4 Molecular docking

Molecular docking of the selected ligands with targeted proteins for DM and associated complication was assessed using PyRx software by AutoDock Vina for molecular docking (https://pyrx.sourceforge.io/) (Scripps Research Institute, La Jolla, CA, USA). To prepare all protein and ligand files for docking and for the generation of docking, parameter input files were incorporated (docking Graphical User Interface frontend PyRx version 0.8).

PyRx was employed to convert all protein and ligand PDB files into PDBQT format. Protonation states for titratable side chains of the protein were based on using OpenBabel (OpenEye Scientific Software, Santa Fe, NM, USA) at pH 7. OpenBabel was used to run energy minimization for selected molecules. Docking boxes were set using the “maximize” option in PyRx around the protein receptor to enable blind docking where the entire protein surface and inner compartments were made accessible for potential binding of ligands (21, 22), initiated by blind docking run.

#### 2.2.5 Molecular dynamics simulation

The docked complex was subjected to dynamics studies to monitor the stability of the protein-ligand complex. GROMACS 4.0.6 software package was used by converting PDB to gmx files followed by generating topology files, defining box, solvating protein, addition of ions, and energy minimization with respective commands. The position was restrained, and molecular production step was run for 100 ns MD simulation. Finally, generating the root-mean-square deviation (RMSD) and root-mean-square fluctuation (RMSF) graphs.

### 2.3 Maintenance of cell lines

INS1 832/13 cells (kind gift from H. Mulder, Malmö) were cultured in RPMI 1640 (Invitrogen, India) supplemented with 10% fetal bovine serum, streptomycin (100 μg/ml), penicillin (100 μg/ml), sodium pyruvate (1 mM), and 2-Mercaptoethanol (50 μM) with 11 mM glucose. The cells were maintained at 37°C with 5% CO_2_ atmosphere incubator.

### 2.4 Cell viability assay

Cell viability analysis was carried out as described by Alley (23). INS-1 cells were seeded in a 96-well microtiter plate followed by incubation at 37°C, 5% CO_2_ for 24h. The spent media were changed, cells were treated with concentrations ranging from 0.1 to 1 µM of pheophorbide A and incubated for 24h and 48h. Media were aspirated from the 96-well microtiter plate, 200 µl of medium containing 10% MTT (3-[4,5-dimethylthiazol-2-yl]-2,5 diphenyl tetrazolium bromide) reagent was then added to each well to get a final concentration of 0.5 mg/ml, and the plate was incubated at 37°C, 5% CO_2,_ for 4h. The medium was removed keeping the formed crystals intact, 100 µl of DMSO was added, and the plate was shaken to solubilize the formed formazan. Absorbance was read at 570 nm (23). The percentage of cell viability was calculated using the following formula:


$$\text{\% Cell Viability}=\frac{\text{Absorbance of treated cells}-\text{ background absorbance }}{\text{Absorbance of untreated cells}-\text{background absorbance }}\times 100$$

### 2.5 Transfection and treatment

Transient transfections were performed by seeding cells on 22-mm poly-L-lysine–coated coverslips with GLUT1–enhanced green fluorescent protein (eGFP) plasmid (Addgene plasmid # 18729; http://n2t.net/addgene:18729; RRID: Addgene_18729) using jetPRIME®. The transfection media were changed to 20 mM glucose and 3 mM glucose after 6h. 500 µl of 0.5 mM of pheophorbide A and 0.5 mM metformin was added and incubated for 14h to 18h. Followed by imaging using total internal reflection microscopy (TIRF-M).

#### 2.5.1 Microscopy

Cells were imaged using TIRF-M based on a Nikon Ti2 Eclipse with a 100×/1.45 objective (Nikon). Excitation was done by DPSS laser at 488 nm. The emission light was captured on an EMCCD camera (Hamamatsu ORCA-Flash 4.0). Scaling was maintained at 130 nm per pixel. Most cells were imaged at 100 ms exposure time.

#### 2.5.2 Image analysis

Granule density of GLUT1 at the plasma membrane of the cell was calculated using “find maxima” function in ImageJ software (NIH; http://rsbweb.nih.gov/ij) (24). GLUT1 trafficking was analyzed using the trackmate function in ImageJ software with 0.3-µm blob diameter.

### 2.6 Glucose uptake assay (glucose oxidase-peroxide enzyme assay)

To assess the effect of pheophorbide A on glucose uptake, a glucose oxidase-peroxide (GOD-POD) assay was carried out using a glucose assay kit (E-BC-K234-S, Elabscience Biotechnology). The GOD-POD assay measures glucose levels within a detection range of 0.05–30 mM, demonstrating a sensitivity of 0.05 mM. The cells were seeded into a 12-well plate at a density of 75,000 cells per well. After 24h, the media were changed to 20 mM glucose and 3 mM glucose, respectively. Followed by treatment of cells with pheophorbide A and metformin at 0.5 mM concentration, respectively, the supernatant (10 µl) was then collected after 18h. The supernatant was incubated with GOD-POD reagent (1,000 µl) for 25 min at 37°C followed by which absorbance was read at 505 nm. A standard curve of 0–30 mM was concurrently plotted with the test samples (16).

### 2.7 Statistical analysis

Values are represented as mean ± SE. Significant differences were analyzed using analysis of variance (ANOVA) by Dunnett’s multiple comparisons test *p*< 0.05, GraphPad Prism 9. Figure were prepared using GraphPad Prism 9 and CorelDRAW.

## 3 Results

### 3.1 *In-silico* methods to assess the potential of pheophorbide A in modulating GLUTs

Pheophorbide A is a bioactive compound that was identified in the polyherbal formulation MA using LC-MS/MS and has been reported to have anti-diabetic activity. However, it is also crucial to assess the drug-like state of bioactive compounds in the human body. During the first phases of drug development, *in-silico* methods were proven to be useful and cost-effective (21). As a result, we used *in-silico* methods to assess the compound’s drug-like, pharmacokinetics, toxicology profile, binding potential, and stability with our target of interest, that is, class I GLUTs.

#### 3.1.1 Toxicology of pheophorbide A

**Figure 1:**
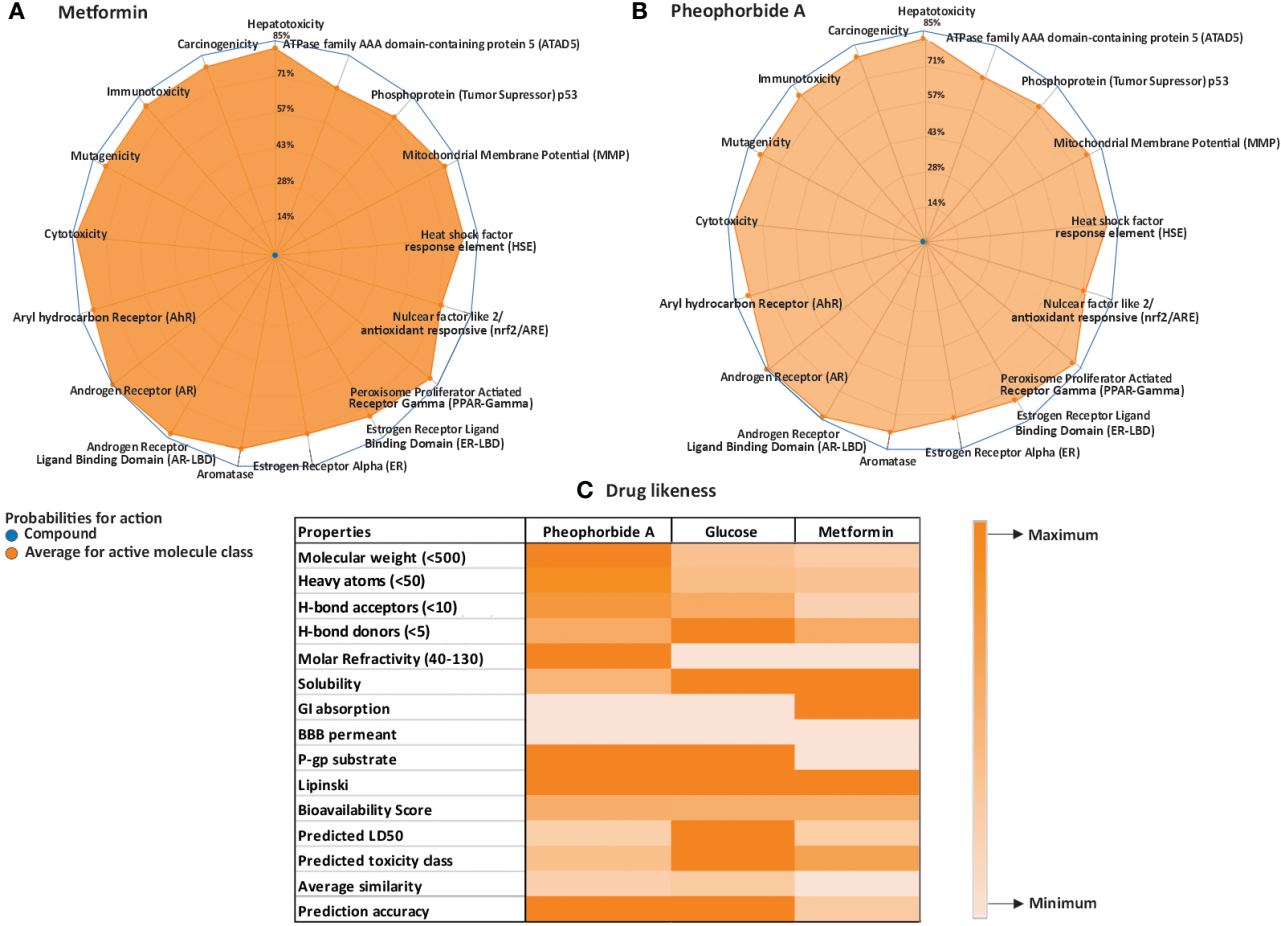
**(A–C)** Physicochemical analysis of the selected compounds; **(A, B)** ProTox ii–generated radar plot for pheophorbide A and Metformin; **(C)** heatmap for Lipinski and SwissADME (Lipinski criteria are mentioned in the properties column, remaining accounts as the maximum and minimum bars represented in the illustration).

Toxicity evaluation is critical in human health and the future drug candidates. ProTox-II server was used for computer-generated toxicology valuation, which involves assessing various qualitative and quantitative factors of the compound (21). PorTox-II computed the lethal dose of pheophorbide A as 40 mg/kg of body weight. Radar chart (**Figures 1A, B**) shows the qualitative endpoint toxicity analysis including cytotoxicity, mutagenicity, carcinogenicity, immunotoxicity, and hepatotoxicity of pheophorbide A (inactive) and metformin (inactive). Endpoint toxicity analysis also showed the compound is inactive. Pheophorbide A data represented that it is non-toxic with respect to the biochemical pathways included in ProTox-II analysis, that is, aryl hydrocarbon, estrogen receptor, androgen, heat shock factor response element, nuclear factor (erythroid-derived 2)-like anti-oxidant responsive element, aromatase, mitochondrial membrane potential, and tumor suppressor protein.

#### 3.1.2 Lipinski rule and pharmacokinetics for pheophorbide A

Selection of drug candidates primarily depends on the rule of five set by Lipinski, which includes molecular weight less than 500, cLog P lower then 5, polar surface area lower than 140 Å, hydrogen bond donors, and acceptor within 5 and 10, respectively (21, 25). It is also critical to study the pharmacokinetics properties of a drug candidate that includes absorption, distribution, metabolism, and excretion. The Lipinski and ADME properties of the selected compound and standard metformin are represented in a heatmap (**Figure 1C**). We used PubChem and SwissADME web servers to evaluate the compounds drug-like and pharmacokinetic properties. Pheophorbide A showed 4 H-bond acceptor, 3 H-bond donor, topological surface area of 133Å, and cLog *p*-value of 3.82. The molecular weight was computed to be 592.68, thus violating the Lipinski rule.

SwissADME server was used to analyze absorption, distribution, metabolism, excretion, and transportation of pheophorbide A in various organs or circulatory systems to ensure its safety. The selected compound did not show the capacity to cross the blood–brain barrier and had low gastro-intestinal absorption and penetrability to glycoproteins. In addition, its solubility was measured between 0 and 2, that is, soluble.

A graphical representation generated by SwissADME, that is, the BOILED-Egg of the compound is represented in **Supplementary Figure S1**. This graphical illustration allows instinctual evaluation of the compound for their gastrointestinal absorption and brain diffusion properties. It is apparent from **Supplementary Figure S1** that the compounds do not penetrate the brain barrier, as it is outside the yellow region or the yolk and is also considered as PGP+, indicated as blue. SwissADME also showed the bioavailability of the compound as 0.56, which was similar to glucose, that is, 0.55 and signifies drug-likeness of the molecule.

#### 3.1.3 Molecular docking to study the interaction of pheophorbide A with class I glucose transporters

**Figure 2:**
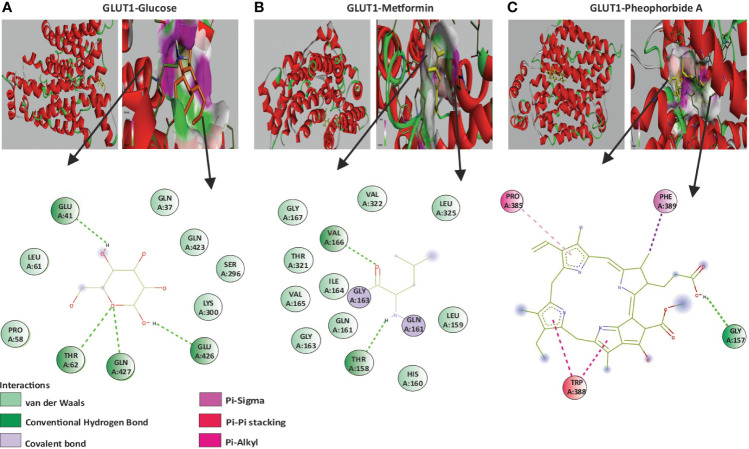
**(A–C)** Molecular docking showing the 2D image of amino acids involved in the binding site and a 3D image of the hydrogen bond interaction of GLUT1 with the ligands; **(A)** GLUT1 and glucose; **(B)** GLUT1 and Metformin; **(C)** GLUT1 and pheophorbide A.

A vital structure-dependent computational technique for predicting theoretical binding mode of various protein ligand is the molecular docking approach. This mainly depends on various crucial characteristics of the receptor/protein of interest and its interaction with the ligand/bioactive compound (22, 26). Considering that pheophorbide A is an anti-diabetic bioactive compound and GSIS being a crucial pathway in DM management, it is important to study pheophorbide A’s interaction with GLUTs, which provide the first point of glucose entry during GSIS. Thus, in this study, we assessed the interaction of pheophorbide A with class I GLUTs, that is, GLUT1–GLUT4. The binding affinity obtained by docking is present in **Table 1**, where the selected compound showed strongest interaction with GLUT4 (−11.2 Kcal/mol) and GLUT1 (−10.7 Kcal/mol), which was also found to be better than the standard drug metformin (−4.9 Kcal/mol). The crucial amino acids involved in the interacting site and the binding forces are depicted in **Figures 2A–C**.

**Table 1:** Binding affinity values using PyRx.

	Pheophorbide A	Metformin	Glucose
GLUT1	−10.7	−4.9	−5.8
GLUT2	−8.7	−4.7	−5.7
GLUT3	−10.1	−4.9	−6.9
GLUT4	−11.2	−5.0	−5.6

#### 3.1.4 Molecular dynamics simulation of pheophorbide A and GLUT1

**Figure 3:**
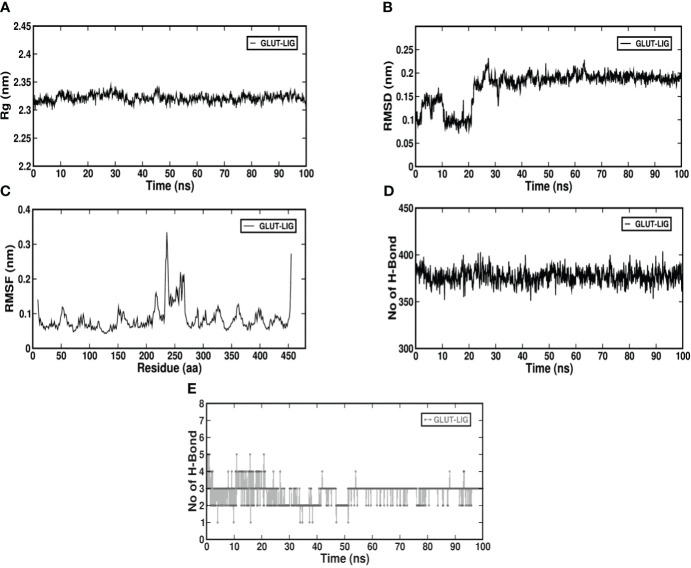
**(A–E)** Molecular dynamics simulation of GLUT1 with pheophorbide A using GROMACS; **(A)** Radius of gyration (Rg) value up to 100 ns; **(B)** root-mean-square deviation of the backbone atom of the complex; **(C)** root-mean-square fluctuation of c-alpha atoms of the complex; **(D)** inter-hydrogen bond of the complex; **(E)** intra-hydrogen bond of the complex.

Molecular dynamics simulation is a computer-oriented biological process related to the size and timescale for assessing the actions of atoms and molecules physically. It is important to recognize the proper functioning of the macromolecules under study, which depends on their mobility. Therefore, validating important details of the protein-ligand association at atomic level is important. Literature suggests expression of GLUT1 to be predominant in β cells when compared to GLUT4 (27); also, **Table 1** shows its binding affinity, which is higher when compared to GLUT2 and GLUT3. Therefore, we studied the stability of the docked complex, that is, pheophorbide A and GLUT1 using GROMACS. The protein-ligand complex stability was investigated over a 100-ns simulation period. **Figures 3A–E** depict the predicted model of pheophorbide A and GLUT1 as revealed by virtual screening. The radius of gyration (Rg) value of the complex was computed to be 2.32 ns and demonstrated that the complex remained very stable in their compact/folded form over a span of 100 ns (**Figure 3A**). To further evaluate the stability of the docked complexes, RMSD plots were generated from the MD simulation of the complex (**Figure 3B**). The backbone of the complex was stable after 20 ns. The plot shows a consistent energy throughout the simulation with an average RMSD value of 0.19 ns. Another vital parameter includes the fluctuations, that is, RMSF (**Figure 3C**), which were observed to be very low, and most of the amino acids were free from RMS fluctuations. Furthermore, there were only a few amino acids, which showed RMS fluctuation at residues 210–260 with the calculated average RMSF of 0.11 nm. **Figure 3D**, which represents the inter-hydrogen bond of the complex, showed stability over a 100-ns run. **Figure 3E** shows the number of hydrogen bonds between the two specified groups at each frame of the simulation, which mostly range between 2 and 4 for pheophorbide A and GLUT1.

### 3.2 *In-vitro* methods to assess the potential of pheophorbide A in modulating GLUTs

As observed from our *in-silico* analysis, pheophorbide A can be considered as a target for GLUT1 binding and a lead molecule for DM management. Additionally, literature also suggests its role in insulin secretion (14); therefore, we assessed its effect on GLUT1 cellular mechanism *in vitro*.

#### 3.2.1 Cell viability

**Figure 4:**
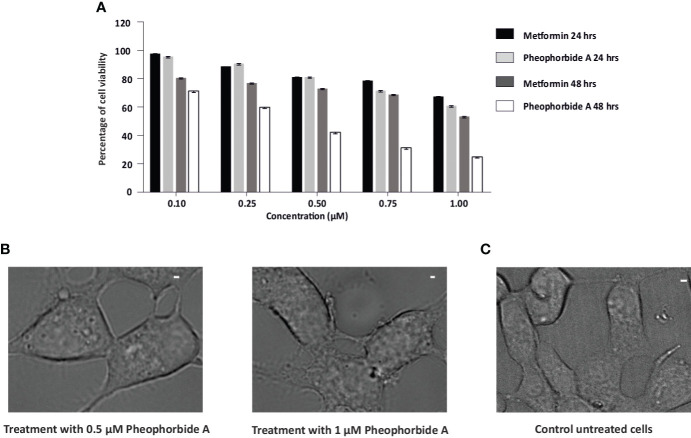
**(A, B)** Effects of pheophorbide A and Metformin on INS-1 cell viability by using MTT assay; **(A)** dose-dependent effect of pheophorbide A and Metformin on the cell viability of INS-1 cells. Data represent the mean ± SE, (*n* = 9; *N* = 3). Statistical analysis using two-way ANOVA by Dunnett’s multiple comparisons test indicates significant differences (****p< 0.0001). **(B)** Representative bright-field microscopy images illustrating the influence of pheophorbide A at concentrations of 0.5 µM and 1.0 µM on INS-1 cells. **(C)** Representative bright-field microscopy images of INS-1 cells under non-treatment conditions. For all images, scale bar is 1 µm.

A vital criteria for drug assessment is determination of a concentration that is not only safe but also attains maximum pharmacological activity of any lead bioactive compound or formulation (28). Therefore, to determine the non-toxic concentration of pheophorbide A and to compare its viability with the standard metformin, MTT analysis was performed. Effect of pheophorbide A and metformin on INS-1 cell viability was evaluated at doses ranging from 0.1 to 1 µM for a span of 24h and 48h. Quantification of formazan estimates the viable cells, which was found to be nontoxic for concentration up to 0.5 µM, with 81.21% viable cells, which was equivalent to metformin (81.08%) (**Figure 4A**). The phase-contrast images in **Figures 4B, C** also show the effect of pheophorbide A on the cell’s integrity at 0.5 µM concentration. The calculated IC_50_ value of pheophorbide A is 1.304 µM/ml at 24h (metformin 1.606 µM/ml at 24h), which decreased to 0.445 µM/ml at 48h. Based on the cell viability assay, the concentration of pheophorbide A of 0.5 µM was considered further for studying its effect on GLUT1 because, at a higher concentration and longer duration, there was little deleterious effects observed on INS1 cells.

#### 3.2.2 Effect of pheophorbide A on GLUT1 density

**Figure 5:**
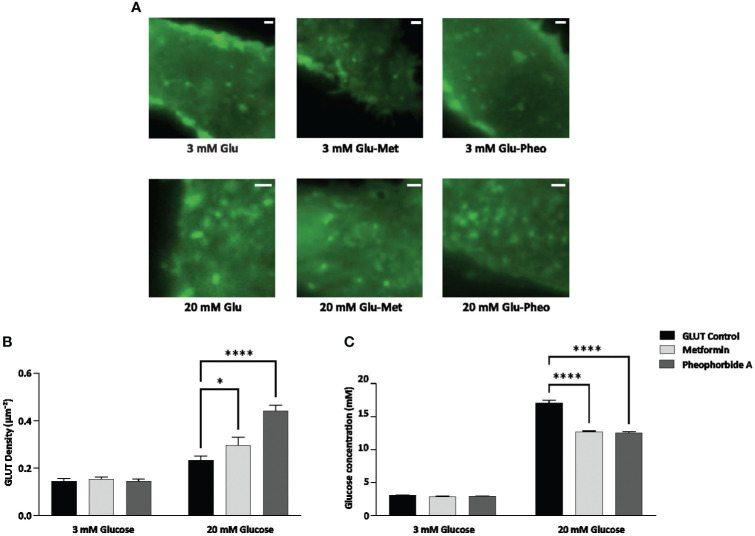
**(A–C)** Effect of pheophorbide A and Metformin on density of GLUT1 at the plasma membrane and uptake of glucose under 3 mM and 20 mM glucose concentration in INS-1 cells; **(A)** GLUT1 density at the plasma membrane, images were taken at 100× using TIRF-M; **(B)** bar graph representing density of GLUT1 with respect to pheophorbide A and Metformin treatment (*n* = 14; *N* = 3); **(C)** bar graph representing glucose uptake by INS-1 cells in treated (pheophorbide A/Metformin) and untreated conditions, cultured at 3 mM and 20 mM glucose media (*n* = 9; *N* = 2). Statistical analysis using two-way ANOVA by Dunnett’s multiple comparisons test indicates significant differences (****p< 0.0001; *p 0.0488). For all images, scale bar is 1 µm.

GSIS is a crucial step for maintaining glucose homeostasis and GLUTs play an important role in transportation of glucose, thereby initiating its breakdown and stimulate insulin secretion. According to literature, this process is disrupted with reduced availability and expression of GLUTs in DM, leading to insulin resistance (Pallavi et al., unpublished) (29, 30). Therefore, we assessed the effect of pheophorbide A and metformin on the density of GLUT1 at the plasma membrane of INS-1 cells using TIRF-M. We transfected INS-1 cells with GLUT1-eGFP, then treated the cells with 0.5 µM pheophorbide A and metformin at high glucose (20 mM) and low glucose (3 mM) concentration. The observed punctate at the plasma membrane for different conditions considered for the study is represented in **Figures 5A, B**, which was analyzed using ImageJ. **Figures 5A, B** show that the density of puncta increases from 3 mM glucose concentration to 20 mM glucose concentration. Our data showed that, at 3 mM glucose concentration, treatment with pheophorbide A and metformin has no effect on GLUT1 density. On the contrary, at 20 mM glucose concentration, pheophorbide A showed significant increase in GLUT1 density, which was higher than the metformin, that is, 0.442 ± 0.01 µm^−2^ and 0.296 ± 0.02 µm^−2^, respectively.

#### 3.2.3 Effect of pheophorbide A on glucose uptake

The release of insulin from β cells is tightly regulated by glucose levels in the bloodstream. Entry of glucose via GLUTs is the initial step in GSIS hence we evaluated if the GLUTs localized at the plasma membrane (**Figure 5C**) also enhance glucose uptake by β cells. We performed glucose uptake assay using GOD-POD method, which relies on glucose oxidase enzymes converting glucose to hydrogen peroxide. The peroxidase involved in this conversion oxidizes pigments, forming a color complex measured at 505 nm (16). Our results showed a significant increase in glucose uptake at 20 mM glucose concentration when compared to control (**Figure 5C**). Cells cultured in 20 mM glucose media showed 17.06 ± 0.162 mM glucose concentrations, whereas pheophorbide A–treated cells showed 12.01 ± 0.178 mM. Based on the data, we can confirm that pheophorbide A promotes glucose uptake. To study mechanistically how pheophorbide A brings about this effect, we studied its effect of pheophorbide A on GLUT1 by tracking analysis.

#### 3.2.4 Effect of pheophorbide A on GLUT1 trafficking

**Figure 6:**
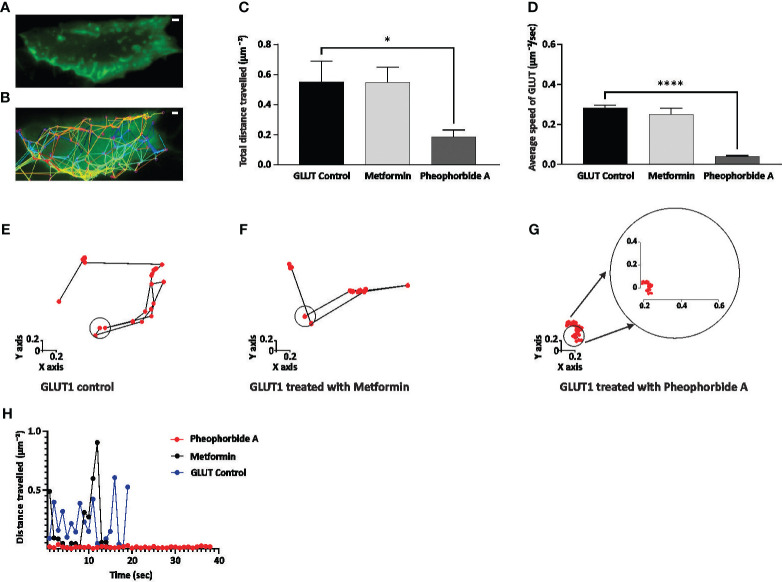
**(A–H)** Effect of pheophorbide A and Metformin on GLUT1 trafficking. Cells were imaged for up to 1 min at 1 s time interval using TIRF-M; the generated tracks were then analyzed; **(A, B)** display of INS-1 transfected cells and their corresponding tracks generated using the TrackMate function in ImageJ; **(C)** quantification of the total distance traveled by GLUT1 puncta in 1 min was calculated (*n* = 15 tracks, *N* = 5 cells); **(D)** calculation of the average speed of GLUT1 puncta observed at 1 s intervals up to 1 min (*n* = 15 tracks, *N* = 5 cells); **(E–G)** depiction of trends observed in GLUT1 under untreated and treated conditions with Metformin/pheophorbide A for single puncta using tracks generated by ImageJ; **(F)** measurement of the distance traveled by a single puncta in untreated and treated conditions (Metformin/pheophorbide A) up to 40 s. Statistical analysis using one-way ANOVA by Dunnett’s multiple comparisons test indicates significant differences (****p< 0.0001; *p 0.0478). For all images, the scale bar is 1 µm. **(F)** During the tracking process, the high mobility of the control blue dot and metformin black dot made accurate tracking challenging, hence, is not represented after 20 s.

We also assessed the trafficking of GLUT1 (**Figures 6A–H**), as it is crucial in understanding glucose uptake. Our study shows that pheophorbide A significantly reduces the total distance traveled by GLUT1 when compared to metformin (**Figure 6C**). **Figures 6E–G** show the GLUT1 puncta movement, where we observe the GLUT1 movement is reduced and is around the cage diameter when treated with pheophorbide A. On the contrary, the movement is away from the cage diameter in control and metformin-treated cells. It is also observed that the speed of GLUT1 puncta was significantly reduced when treated with pheophorbide A (0.039 ± 0.005 µm^−2^/s) in comparison to control (0.28 ± 0.012 µm^−2^/s) and standard drug metformin (0.250 ± 0.032 µm^−2^/s) (**Figure 6D**). Based on the results, pheophorbide A arrests the mobility of GLUT1 transporters, thus promoting its interaction and, hence, promoting glucose transport.

## 4 Discussion

Bioactive compounds, present in natural sources, have been widely considered for drug development in recent times. In the present study, polyherbal formulation MA, a complex concoction of bioactive compounds, was considered, which showed various lead phytocompounds. Our previous study reported pheophorbide A in the major peak of LC-MS/MS of MA (12) similar to other supporting studies in the literature with regard to DM management (14, 15). These studies suggest that pheophorbide A manages DM by enhancing insulin secretion. Literature suggests that islet hormone secretion is regulated by glucose levels in the blood and decreased secretion during diabetes correlates with disrupted secretion of pancreatic hormones such as insulin, glucagon, and somatostatin (31). Secretion of islet hormones in insulin secreting β cells is, in turn, regulated via uptake of glucose through GLUT transporters (32–34). Our previous work showed how insulin secretion is disrupted due to endoplasmic reticulum stress in single β cells during DM (35, 36). Expression of GLUT transporters as well is compromised in case of DM (37, 38); a better uptake of glucose to β cells would help to stimulate GSIS. Considering this, we wanted to explore the effect of pheophorbide A on GLUTs and unveil its effect on glucose stimulation during GSIS. We have accessed the effect of pheophorbide A on GLUTs using *in-silico* and *in-vitro* methods.

One of the crucial criteria in drug development includes assessing the adverse effects that might arise due to multitude of variables, including dose, mode of administration, drug metabolism, allergy, and so forth. This necessitates the use of computational approaches to validate the compounds pharmacokinetics, toxicity, affinity, and stability, which might help to mitigate/manipulate the lead in required form to manage the side effects (39). Our findings demonstrate that pheophorbide A complies with all of the Lipinski’s rules, with an exception to the molecular weight, which is 592.68 instead of the indicated value of fewer than 500. Mahgoub et al. (40) and Tijjani et al. (41) suggest that a compound is considered of high risk if it breaks more than one of the criteria. Additionally, it is also important to note that these rules are not strict cutoffs but rather guidelines to make the drug development process better. Computational studies predict the drug-like and ADME profile of lead drug compound prior their synthesis and clinical trials. This aids in the discovery of the balanced compound during the synthesis process and minimizes the possibilities of failure during the clinical trials while also be economical (42). To predict the balance for the selected compound in the present study, SwissADME computed its limited gastro-intestinal absorption, which might be due to its large molecular weight/PGP+, whereas all other metrics were determined to be satisfactory. The bioavailability of pheophorbide A, at 56%, suggests that a significant portion of the administered dose reaches the bloodstream, allowing for systemic circulation. This indicates its utilization by the body to exert its therapeutic effects. We also computed the toxicology profile using ProTox-II, which showed no toxicity of the compound and the lethal dose as 40 mg/kg of body weight. In contrast to our *in-silico* data, Kim et al. (15) showed that 2 g/kg body weight of pheophorbide A was administered to 4‐week‐old male ICR mice and was found to be non-toxic. Another report suggests that maximum permissible amount of pheophorbide A was determined at 1,000 mg/g and a recommended dosage of 25 mg/day (43).

Application of computer-aided drug design techniques has resulted in the successful development and subsequent Food and Drug Administration approval of various medications. Notable instances include dozamide, approved in 1995; imatinib in 2001; dasatinib in 2006; and ponatinib in 2012, demonstrating the impact of computational methods on pharmaceutical advancements (44). Considering this, we evaluated pheophorbide A–binding affinity with GLUTs using molecular docking, which showed the highest binding affinity with GLUT4 followed by GLUT1. Previous reports show expression of GLUT1 to be predominant in β cells when compared to GLUT4 (27). Therefore, we access the bond and amino acids involved in the interaction site between pheophorbide A and GLUT1. Pheophorbide A and GLUT1 showed an essential and strong covalent hydrogen bond with GLY157, while double-bond pi-pi stacks with TRP388, pi-alkyl with PRO385, and Pi-sigma with PHE389. On the contrary, majority of the bonding of GLUT1 and metformin is weak van der Waals forces. This might explain the higher binding affinity of pheophorbide A with GLUT1, which might be due to the chemical bond formation, that is, covalent and non-covalent interactions, which provide better binding affinity when compared to van der Waals forces (45, 46). Literature showed presence of the amino acid TRP388 in the interreacting site with various phytochemicals, namely, protocatechuic acid, cafeic acid, 3-o cafeoylquinic acid, rutin, dicaffeoylquinic acid, dicaffeoylquinic acid, chlorogenic acid, luteolin, alstonin, and the standard drug glibenclamide, thus might be considered as a crucial site for binding (47–49). Our results also showed the presence of GLY in metformin as well as pheophorbide A and was also previously reported in glibenclamide (47). Computational structural biology and chemistry suggests understanding the dynamic behavior of a ligand-protein complex in a stimulated environment as biological system are not static (50). Therefore, our present study further evaluates the stability of the complex GROMACS was used where RMSD and RMSF are crucial in understanding the behavior of biomolecular systems during simulations. Previous reports use these metrics to assess the stability of protein structures, identify flexible or stable regions within the proteins, and gain insights into the dynamics and conformational changes of biological macromolecules (46, 51). Considering this, our study showed low values for RMSD and RMSF showing stability of the complex, followed by Rg of the protein, which determines the compactness of the complex. Therefore, a stably folded protein would most likely maintain a relatively constant Rg. When a protein unfolds, its Rg changes over time (50). Our data showed that the complex under study was stable. We also observed that the amino acid involved in the interacting/binding site are not present in the fluctuation range of amino acid residues, hence supporting a stable complex hypothesis.

*In-silico* investigations have showed promising results, indicating that the examined compound may be effective. Thus, we initiated *in-vitro* evaluations, with the MTT assay, to validate these findings and further assess the biological activity. A rapid approach for assessing cell viability and proliferation was developed by Mosmann (52). It involves analyzing the reduction of MTT reagent to formazan, which is correlated with the amount of metabolically viable cells (53). Our findings showed that the INS-1 cell viability of pheophorbide A and metformin was equivalent up to 1.00 µM concentration at 24h, but only up to 0.25 µM at 48h. Our data corroborate with previous results, which show pheophorbide A non-toxic concentration to be 1 μM in mouse murine β-cell line—MIN-6 cells (14). Furthermore, an earlier report on 3T3-L1 cell also showed pheophorbide A to be non-toxic up to 2,082.5 μM concentration (15). Cell viability of a compound may vary with different cell line, which is evident to evaluate its effect on INS-1 cells. The observed cell viability for INS-1 cells helps to confirm that the compounds are safe for cells, laying the groundwork for continued research and development for assessing their effect on GLUT1.

Management of DM has a cardinal step, that is, maintaining glucose homeostasis where GLUT1 plays an important role in human β cell for glucose uptake and insulin secretion (54, 55). Previous study reports metformin effect on expression of glucose transports (GLUT1 and GLUT4) in heart and muscle cells, on diabetic male Wistar rats (streptozotocin), which showed that metformin significantly enhances glucose uptake at high concentrations, independent of insulin. Therefore, suggesting that this effect might be due to a redistribution of glucose carriers from inside the cell to the cell membrane (56), Sokolovska et al. (57) suggested an increase in GLUT1 expression in the liver, kidney, muscle, and heart tissues of rats. Therefore, literature suggests that drug development targeting reduction of glucose levels or enhancing insulin levels is available for DM management, lacking their mechanistic effect on GLUT1 (58). Considering the importance of GLUT1 transporter specifically in human β cell assessing its effect might open new aspect in managing DM. Therefore, to assess the effect of pheophorbide A mechanistically in trafficking of GLUT1 transporters to aid better understanding of glucose uptake, we transfected INS-1 cells with GLUT1 labeled with eGFP. The observed data in the present study showed a significant increase in GLUT1 density at 20 mM glucose concentration when compared to control at the same glucose concentration. It is also important to note that we observed no change in GLUT1 density at 3 mM glucose concentration with respect to control or treatment with pheophorbide A/metformin. High density of GLUT1 in the plasma membrane of the cell at 20 mM glucose concentration may suggest active and efficient glucose transport mechanism thus might enhance insulin secretion and maintain glucose homeostasis in DM (56, 59, 60).

The presence of pheophorbide A increased the uptake of glucose in synchronization with higher density of GLUT1 at the plasma membrane of β cell. Our results support previous data, which suggest that increase number of GLUT4 on adipose and muscle cells correlates with increased glucose uptake (61) in those tissues.

We also evaluate if the turnover of GLUT1 transporters was affected, by assessing the movement of GLUT1 at the plasma membrane in presence of pheophorbide A compared with control. Surprisingly, there was a reduced mobility of GLUT1 transporters in presence of pheophorbide A shown by reduced distance traveled of turnover of GLUT1 transporters at the plasma membrane. Literature suggests ligand and protein interaction modifies/influences the movement of protein by various methods one of which is by stabilizing the complex and may improve its functional activity by locking it in a certain shape that is advantageous to its function in cellular activities (62–64). Literature also suggested a mutation that alter the expression/amino acid sequence of GLUTs affects the movement of GLUT leading to altered glucose uptake and efficiency under varying physiological conditions (65). Brownian movement of a protein influences its molecular interactions and diffusion, which plays a vital role in drug development. It impacts receptor-ligand binding, influences drug transport within biological systems, and is crucial in *in-silico* modeling for predicting molecular behavior assed in molecular dynamics simulation. In line with this we infer that the better binding of pheophorbide A with GLUT1 is arresting its mobility at the plasma membrane. Our *in-silico* data further provide proof showing strong binding affinity of pheophorbide A and GLUT1 as well.

The main objective of the present study was to determine if GLUT1 transporter efficiency can be improved with addition of pheophorbide A in β cell with a possibility of using pheophorbide A to stimulate GSIS during DM. Therefore, we assessed the binding affinity, stability, density, and movement of GLUTs with pheophorbide A. Our data also showed strong binding affinity and stability of the complex. We observed that pheophorbide A further potentiates glucose uptake, which may lead to insulin secretion reported in previous studies (11, 13). In every metric, pheophorbide A showed better efficiency of binding with GLUT1 to improve glucose transport. This opens up the possibility of using pheophorbide A supplements to increase glucose uptake into β cells to stimulate GSIS overall aiding in better management of DM.

## Data availability statement

The original contributions presented in the study are included in the article/**Supplementary Material**. Further inquiries can be directed to the corresponding author.

## Ethics statement

Ethical approval was not required for the studies on animals in accordance with the local legislation and institutional requirements because only commercially available established cell lines were used.

## Author contributions

NG: Conceptualization, Data curation, Formal analysis, Funding acquisition, Investigation, Methodology, Project administration, Resources, Software, Supervision, Validation, Visualization, Writing – original draft, Writing – review & editing. SP: Conceptualization, Data curation, Formal analysis, Investigation, Methodology, Resources, Software, Supervision, Validation, Writing – original draft, Writing – review & editing. AP: Data curation, Formal analysis, Methodology, Writing – review & editing.
