# Supplementary material for: Exploring the potential of pheophorbide A, a chlorophyll-derived compound in modulating GLUT for maintaining glucose homeostasis
Source: Front Endocrinol (Lausanne). 2024 Mar 11;15:1330058. doi: 10.3389/fendo.2024.1330058 (PMC10961331; doi:10.3389/fendo.2024.1330058)
Supplement: Supplementary file 1 [file Image_1.pdf]

Supplementary Material

1 Supplementary Figure

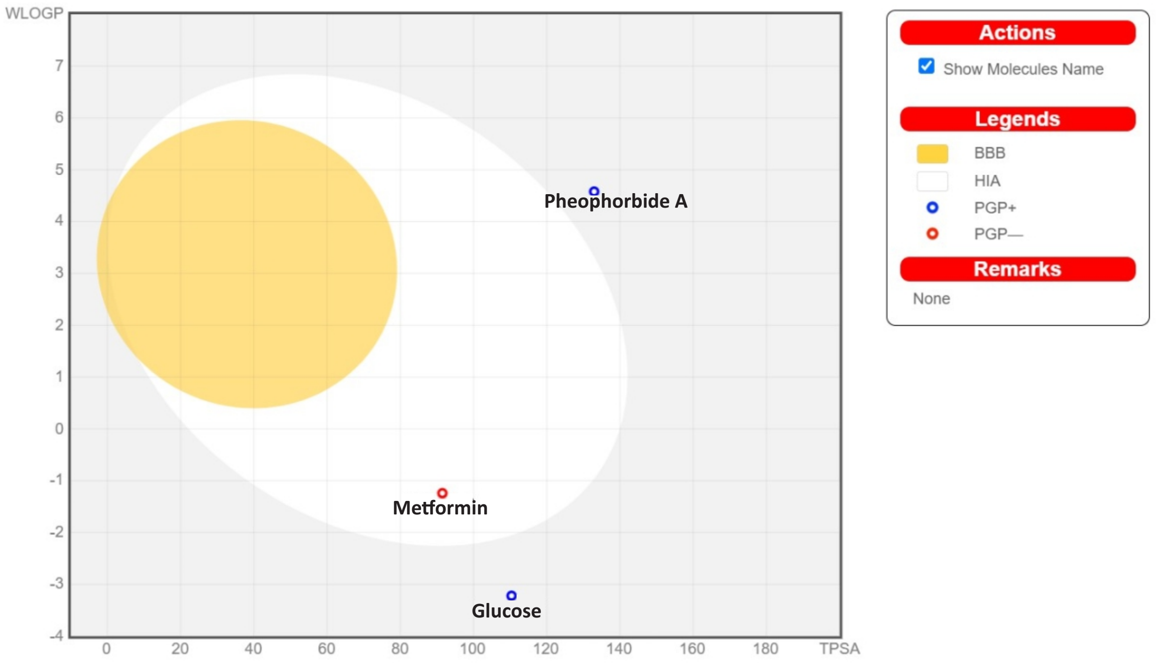

Figure 1.S BOILED-Egg for the selected compound represented as WLOGP-versus-TPSA generated by SWISS-ADMET
